# GIS-Based Temporal and Spatial Analysis of Industrial Wastewater Pollution in the Konya Municipal Sewer System

**DOI:** 10.3390/molecules31101738

**Published:** 2026-05-19

**Authors:** Süheyla Tongur, Sefa Çetin

**Affiliations:** 1 Department of Environmental Engineering, Faculty of Engineering and Natural Sciences, Konya Technical University, 42250 Konya, Turkey; 2 Koski General Directorate, 42060 Konya, Turkey

**Keywords:** wastewater, wastewater treatment, sewer monitoring, sewer system, ArcGIS

## Abstract

Wastewater containing high pollutant loads is discharged into the municipal sewerage system by industrial facilities operating within the industrial zones of Konya, Türkiye. Although regulations mandate that wastewater be treated to comply with specified discharge standards, some facilities lack pretreatment systems due to high capital and operational costs, while existing systems experience operational deficiencies. As a consequence, operational disruptions and increased environmental risks occur within the municipal sewerage system. Periodic sampling and inspection activities conducted by municipal authorities are becoming increasingly challenging for effective monitoring and evaluation as the number of facilities increases. In this study, a Geographic Information System (GIS)-based approach was developed to enhance monitoring effectiveness, and industrial wastewater quality data were analyzed using ArcGIS Pro 2.9 software (Esri, Redlands, CA, USA) to generate spatial pollution distribution maps. Samples were collected from five industrial facilities and four sewer junction points located in the Hacıyusufmescit, Emirgazi, and Fetih neighborhoods, where odor problems are frequently reported, during the 2022–2023 period. It was determined that COD (24,960 mg/L), BOD (2970 mg/L), and oil and grease (254 mg/L) concentrations significantly exceeded the regulatory discharge limits, particularly during the summer season. The results demonstrate that GIS-based monitoring systems constitute an effective tool for the early detection of pollution and odor-related problems at the urban scale, for the systematic management of control processes, and for the facilitation of evidence-based decision-making.

## 1. Introduction

With the acceleration of industrialization and urbanization processes, the volume of wastewater generated by industrial activities and the associated pollutant load have increased significantly. The application of appropriate pretreatment to industrial wastewater prior to its discharge into the municipal sewerage system is critical for protecting sewerage infrastructure and ensuring the effective operation of municipal wastewater treatment plants. The discharge of industrial wastewater into the municipal sewerage system without adequate pretreatment and without compliance with discharge limit values causes physical and chemical deterioration of sewer lines and reduces treatment efficiency by inducing sudden hydraulic and organic load increases in municipal wastewater treatment plants [1]. This situation adversely affects infrastructure performance and poses significant risks to public health and environmental sustainability. Industrial facilities are regularly inspected by municipal authorities to ensure the proper operation of sewerage systems and to prevent load fluctuations in wastewater treatment plants. Within this framework, wastewater samples are collected from facility discharge points and pretreatment outlets at intervals specified by the applicable legislation. However, this monitoring approach is largely limited to facility-based assessments and does not adequately reveal the spatially integrated performance of the sewerage system. Therefore, integrated database systems that enable process-based and spatial monitoring of the sewerage system are required in addition to facility-based monitoring. Currently, many cities manage data collection, storage, updating, analysis, control, and visualization processes in an integrated manner through Geographic Information System (GIS)-based applications. In this context, Konya Metropolitan Municipality aims to enhance service efficiency, responsiveness, and quality by expanding GIS-based data integration across various service areas. Similarly, spatial data related to infrastructure components, including sewer networks, drinking water distribution lines, freshwater supply lines, reservoirs, and wells within KOSKİ, are recorded in the GIS environment. The GIS database contains technical data on the municipal sewerage system, including manholes, manhole covers, pipe diameters, pipe materials, and elevation data. However, data related to the physicochemical quality characteristics of the wastewater conveyed by the system have not yet been integrated into this database structure. Nevertheless, comprehensive and process-based monitoring of such data can be achieved through the existing GIS infrastructure. Accordingly, in this study, the sewerage system within an area characterized by high industrial density was monitored as a pilot region, and the analytical results were transferred to ArcGIS Pro 2.9 software (Esri, Redlands, CA, USA) for spatial evaluation.

### 1.1. Classification of Wastewater and the Use of GIS in Municipalities

#### Definition and Classification of Wastewater

Wastewater refers to water whose physical, chemical, or biological characteristics have been altered as a result of domestic, industrial, and commercial activities following its use. Depending on the source, the composition of wastewater varies considerably, with pollutant types and concentrations differing according to usage patterns. According to a broader definition, wastewater includes not only water generated by residential, commercial, institutional, and industrial activities but also water that becomes surface or subsurface runoff following precipitation events from streets, parking lots, and similar urban surfaces [2,3].

In regulatory frameworks, wastewater is defined as water that has been contaminated or whose properties have been altered as a result of domestic, industrial, agricultural, or other activities, and its discharge into the municipal sewerage system is subject to specific criteria and restrictions [4]. These definitions provide a fundamental framework for wastewater classification, determination of treatment requirements, and management of environmental impacts.

**Table 1:** Discharge standards for industrial wastewater discharged into the municipal sewerage system [1,4].

Parameters	Sewage Systems Wastewater Infrastructure Facilities Resulting in Biological or Equivalent Treatment (2 h Composite Sample)	For Facilities with a Wastewater Flow Rate ≤ 5m^3^/Day (2 h Composite Sample)	For Facilities with Wastewater Flow Rates > 5 m^3^/Day ≤ 50 m^3^/Day (2 h Composite Sample)
Temperature (°C)	40	40	40
pH	6–10	6–10	6–10
Conductivity (mS)	4.000	6000	5000
Suspended solids (SS) (mg/L)	400	1000	600
Oil and grease (mg/L)	150	300	200
Tar and petroleum-based oils (mg/L)	50	120	75
Chemical oxygen demand (COD) (mg/L)	800	4000	1800
Phenol (mg/L)	20	30	25
Sulfate (SO_4_^2−^) (mg/L)	1700	2200	2000
Arsenic (As) (mg/L)	3	10	5
Total lead (Pb) (mg/L)	3	10	5
Total mercury (Hg) (mg/L)	0.2	0.4	0.3
Total cadmium (Cd) (mg/L)	2	5	4
Total cyanide (CN^−^) (mg/L)	10	20	15
Total chromium (Cr) (mg/L)	5	10	7
Free chlorine (mg/L)	5	10	7
Total sulfur (S) (mg/L)	2	5	4
Total copper (Cu) (mg/L)	2	5	4
Total nickel (Ni) (mg/L)	5	10	7
Total zinc (Zn) (mg/L)	10	20	15
Total tin (Sn) (mg/L)	5	10	7
Total silver (Ag) (mg/L)	5	10	7
Total iron (Fe) (mg/L)	5	10	7
Total aluminum (Al) (mg/L)	5	10	7
Chloride (Cl^−^) (mg/L)	10.000	15.000	12.000

### 1.2. Use of Geographic Information Systems (GISs) in Municipalities

**Figure 1:**
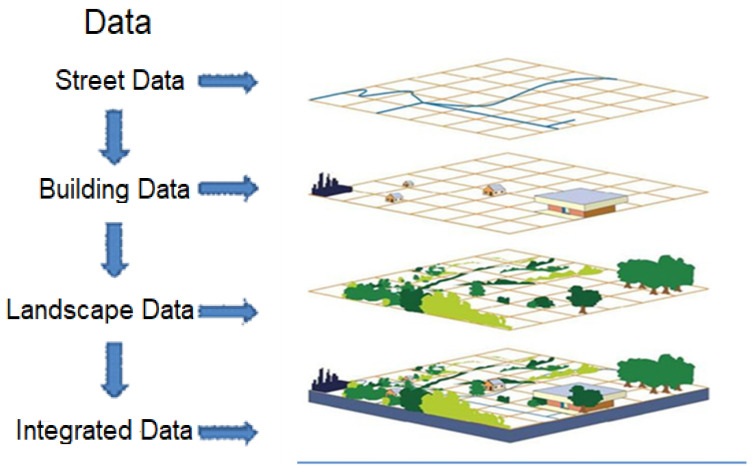
GIS data layers [1].

Geographic Information Systems (GISs) are integrated decision-support systems that enable the collection, computerized storage, updating, management, analysis, and visualization of data related to the Earth’s surface for specific purposes. GIS provides an analytical framework for decision-making processes by revealing the spatial distribution and interrelationships of location-related events. These systems contribute to the effective maintenance of geographic records, the development of location-based management applications, and cost reduction through improved operational efficiency [1,5,6]. GIS is a computer-based system used to obtain, store, verify, and visualize data related to locations on the Earth’s surface, enabling the creation, management, analysis, and mapping of spatial data. These systems provide a powerful infrastructure for map-based analysis by integrating location data with other types of descriptive data. GIS has a wide range of applications, from scientific research to industrial practices, facilitating the understanding of spatial patterns and relationships and thereby improving communication, management, and decision-making processes. Today, numerous organizations widely use GIS-based mapping applications to perform analyses, share information, and generate solutions to complex problems [7,8]. The core functions of GIS encompass data management, analysis, updating, quality control, scheduling, and visualization processes. Early studies on GIS functions identified list management, data collection, data entry and storage, analysis, information generation, basic subsystems, and information utilization as key components [9,10,11]. Subsequent studies expanded this framework by including management and information utilization processes among the system components [11] and emphasized that quality control mechanisms constitute an integral component of GIS functions [12]. GIS consists of four fundamental elements: the creation of geographic data, database management, analytical modeling, and map-based visualization. The system structure generally comprises data, hardware, and software components. GIS can integrate different data layers using spatial location as a primary reference and bring together multisource information on a single platform. Cartographic data (e.g., rivers, roads, and topographic features), photographic data, digital datasets, satellite imagery, remote sensing outputs, field survey data, and electronic tables are among the data types that can be integrated into GIS [13]. The transfer of these data into the system is defined as the “data capture” process. Satellite and unmanned aerial vehicle imagery obtained through remote sensing technologies enable a wide range of data, from land use to environmental changes, to be integrated into the GIS environment [14]. Furthermore, tabular information, such as population demographics, socioeconomic indicators, and behavioral data, can also be integrated into the GIS database [15]. The fundamental strength of GIS technology lies in its ability to analyze multidimensional data obtained from different sources by overlaying them as layers on a single map [16]. Location information serves as the primary reference variable that integrates seemingly unrelated datasets [17]. This capability enables GIS to examine spatial relationships in a multidimensional and integrative manner. For example, a city’s road network, building inventory, topographic features, and land use data can be analyzed within the same spatial framework, with different data layers added or removed as needed (Figure 1) [18,19]. This integrated analytical capability makes GIS an indispensable tool for location-based planning, monitoring, and decision-support processes [20].

## 2. Results and Discussion

### 2.1. Urban Information System in Konya

The establishment of the Urban Information System in Konya dates back to 2015. Within this framework, the Metropolitan Municipality has shared technical, social, and economic data related to the city through GIS-based platforms. As a result, the municipality has enhanced service efficiency, responsiveness, and quality and continues its efforts to ensure that more accurate decisions are made based on up-to-date data [1].

**Figure 2:**
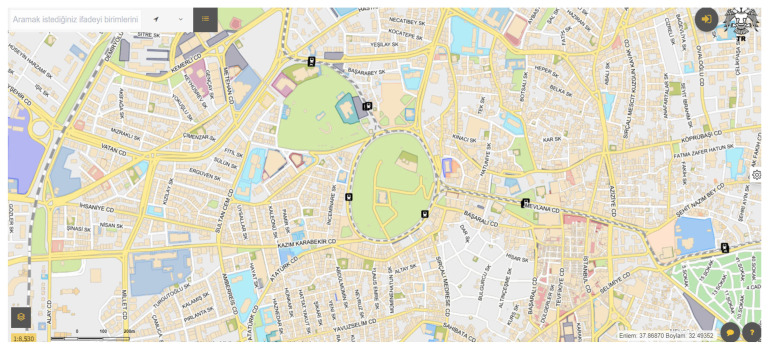
Konya Urban Information System [1].

**Figure 3:**
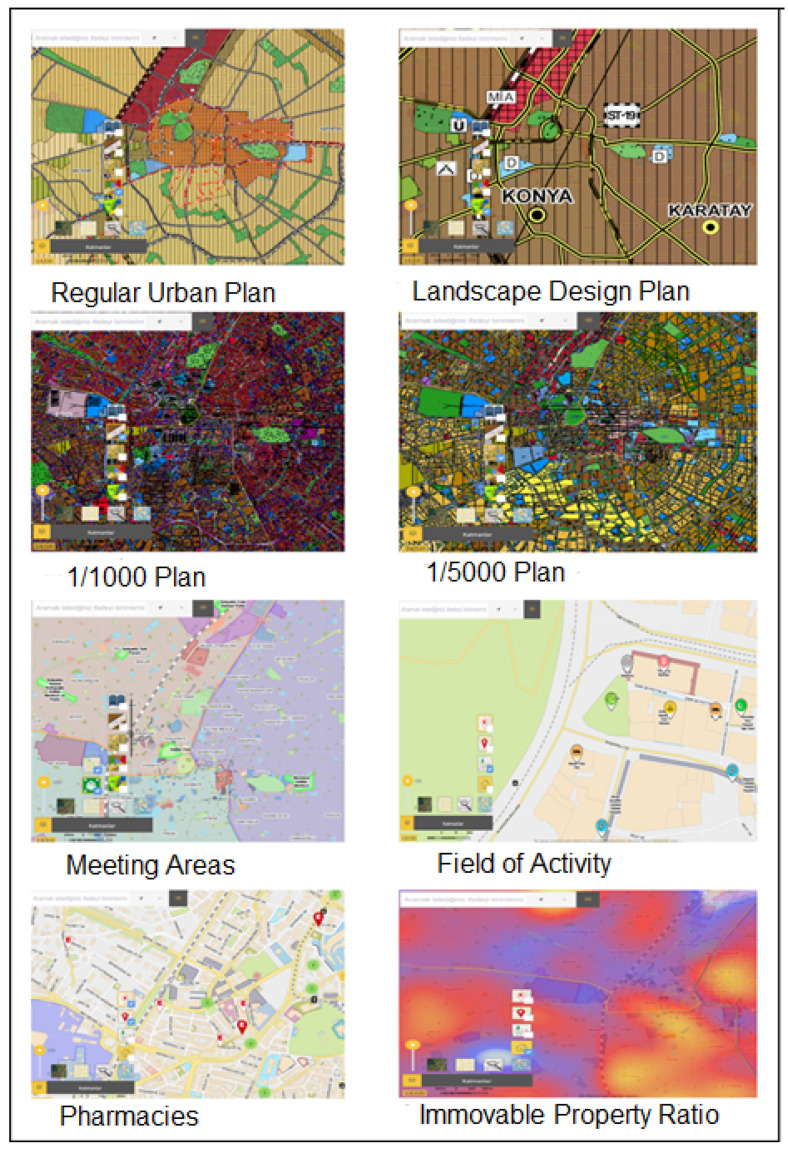
Konya City Information System Layers [1].

The Konya City Information System includes the following modules: Location Display, Nearest Facility, Layer Tree, Building Information, Route Analysis, Construction Permit, Block Parcel, Registered Building Information, Content Management, Taxi Stand, Pharmacy, and Measurement Tools (Figure 2 and Figure 3).

Smart city applications in Konya are implemented through various functional components within the Urban Information System infrastructure.

Smart city applications in Konya appear as different applications through specific components.

### 2.2. Sample Point Analyses

The analytical results of samples collected from five industrial facilities located in the Hacıyusufmescit, Emirgazi, and Fetih neighborhoods of Konya province are presented in Table 2, Table 3, Table 4, Table 5 and Table 6. An examination of Table 2 and Table 3 indicates that the results obtained from Industry 1 and Industry 2 comply with the discharge standards specified in the KOSKİ Wastewater Discharge into the Sewerage System Regulation. It was determined that these facilities implement pretreatment prior to discharging wastewater into the municipal sewerage system. Additionally, the pH values measured during the winter and spring seasons at Industry 1 were found to fall within the specified regulatory range.

Table 4 indicates that the suspended solids (SS) concentration measured during the summer season exceeded the regulatory limit specified in Table 1. However, the pH value for the same period was determined to fall within the threshold-adjacent range.

Table 5 indicates that the pH, COD, and SS concentrations for Industry 4 during the summer season exceeded the regulatory limits specified in Table 1. A pH level outside the optimal range required for treatment processes reduces the effectiveness of chemicals and polymers used within facility treatment systems, resulting in increased chemical consumption and decreased process efficiency. Similarly, pH levels outside the appropriate range entering the municipal wastewater treatment plant adversely affect microorganisms involved in biological treatment processes, thereby reducing overall biological treatment efficiency. Furthermore, acidic conditions (low pH) increase the risk of corrosion within sewer pipelines.

Elevated COD concentrations increase the oxygen demand in treatment processes, thereby limiting the availability of dissolved oxygen for biological oxidation and adversely affecting overall treatment efficiency. SS concentrations exceeding regulatory limits increase physical pollution loads, elevate the risk of abrasion and mechanical damage to treatment equipment, and reduce process efficiency. In addition, elevated suspended solids can promote sedimentation and sludge accumulation in receiving environments, thereby adversely affecting ecological balance. In sewer systems, insufficient hydraulic flow conditions promote the accumulation of suspended solids, increasing the risk of blockages and localized flooding and consequently raising operational and maintenance costs.

Table 6 indicates that the COD, suspended solids, and oil and grease concentrations for Industry 5 during the summer season exceeded the regulatory limits specified in Table 1. Furthermore, the COD and oil and grease concentrations measured during the spring season were found to exceed these regulatory limits. Oil and grease concentrations exceeding acceptable limits promote the formation of deposits within sewer systems, resulting in pipe cross-sectional narrowing and blockages. In addition, such accumulations increase the risk of mechanical wear and damage to sludge-processing equipment and valves, thereby negatively affecting overall operational performance. The relative resistance of oil and grease components to anaerobic degradation can contribute to foam formation in digesters, thereby reducing biological treatment efficiency.

The analytical results of samples collected from four sewer junction points in the Hacıyusufmescit, Emirgazi, and Fetih neighborhoods of Konya province are presented in Table 7. Examination of the results obtained from the sewer junction points revealed elevated COD and suspended solids concentrations (Table 7). These findings indicate that the wastewater entering the sewer system in these areas contains substantial organic loads and elevated suspended solids concentrations.

### 2.3. Temporal and Spatial Mapping of Analysis Results

These maps cover the Hacıyusufmescit, Emirgazi, and Fetih neighborhoods within the Karatay District of Konya province. Samples were collected from five monitoring points during the winter, spring, and summer seasons of 2023, and spatial distribution maps were generated using the inverse distance weighting interpolation method. Accordingly, the interpolated pH distribution maps were classified according to the following value ranges:

- pH < 6.00—Noncompliant (Red);
- 6.00 ≤ pH ≤ 6.50—Threshold-adjacent (Yellow);
- 6.51 ≤ pH ≤ 8.50—Compliant range (Green);
- 8.51 ≤ pH ≤ 9.50—Threshold-adjacent (Yellow);
- pH > 9.50—Noncompliant (Red).

**Figure 4:**
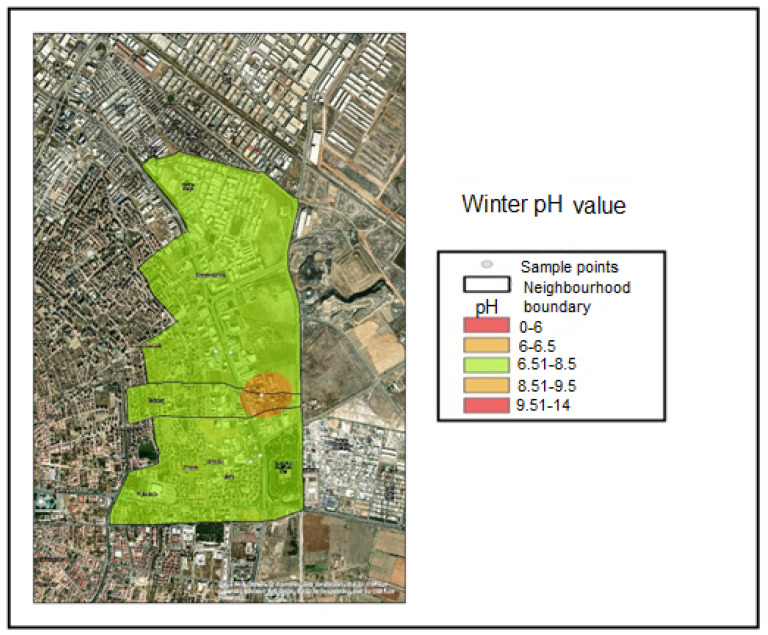
Winter season pH distribution map.

**Figure 5:**
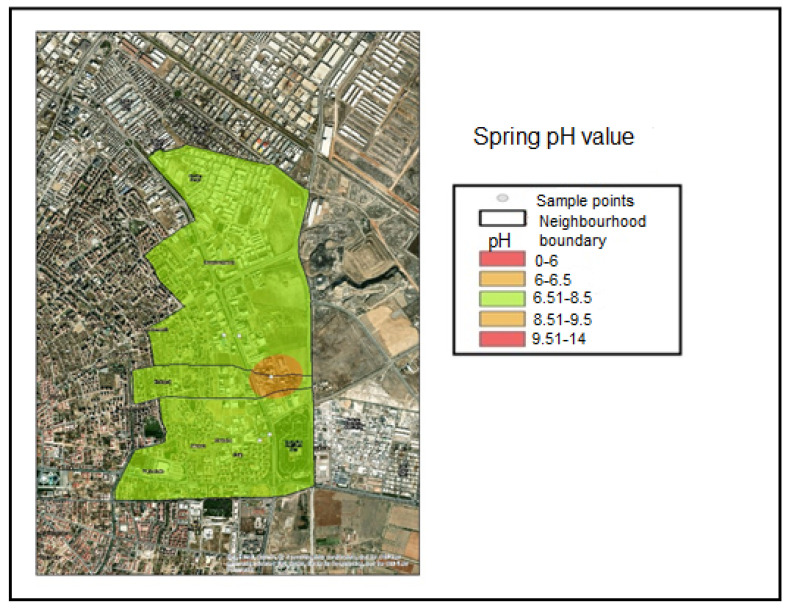
Spring season pH distribution map.

**Figure 6:**
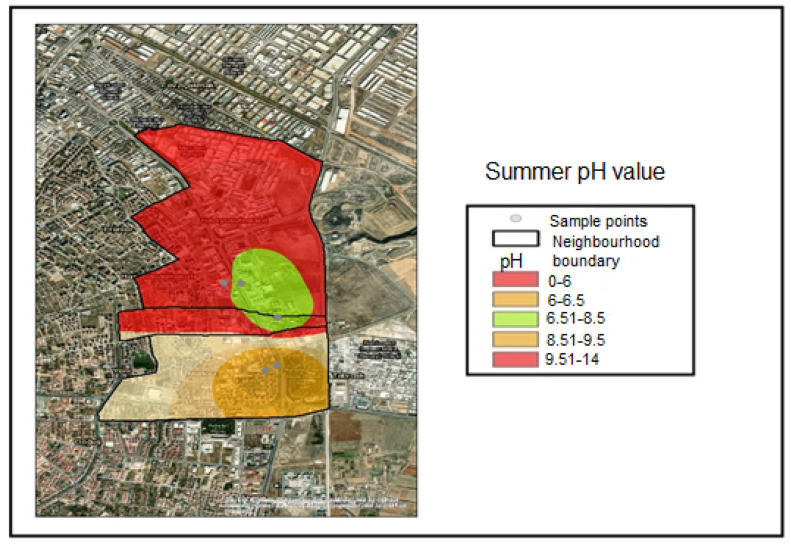
Summer season pH distribution map.

These classification ranges were determined based on the applicable regulatory discharge criteria. Based on the sample results obtained during the winter and spring seasons, only Industry 1 was classified within the threshold-adjacent range (6.00–6.50) and was therefore represented in yellow. This interval corresponds to values approaching the lower regulatory threshold (Figure 4). During the winter and spring seasons, Industries 2, 3, 4, and 5 were classified within the compliant range (6.51–8.50) and were therefore represented in green. This interval represents the compliant pH range defined by the discharge regulation (Figure 4 and Figure 5). Figure 6 indicates that the summer pH values for Industries 1 and 2 remained within the compliant range, whereas Industry 3 fell within the upper threshold-adjacent interval (8.51–9.50) and Industry 5 fell within the lower threshold-adjacent interval (6.00–6.50). Industry 4 exhibited pH values below 6.00, thereby exceeding the lower regulatory limit and being classified as noncompliant (red).

Spatial interpolation was performed within the GIS application for the COD analyses conducted on the collected samples. The COD classification ranges applied in the interpolation analysis were defined as follows:

- COD ≤ 800 mg/L—Compliant range (Green);
- 801–960 mg/L—Threshold-adjacent range (Yellow);
- COD > 960 mg/L—Noncompliant (Red).

**Figure 7:**
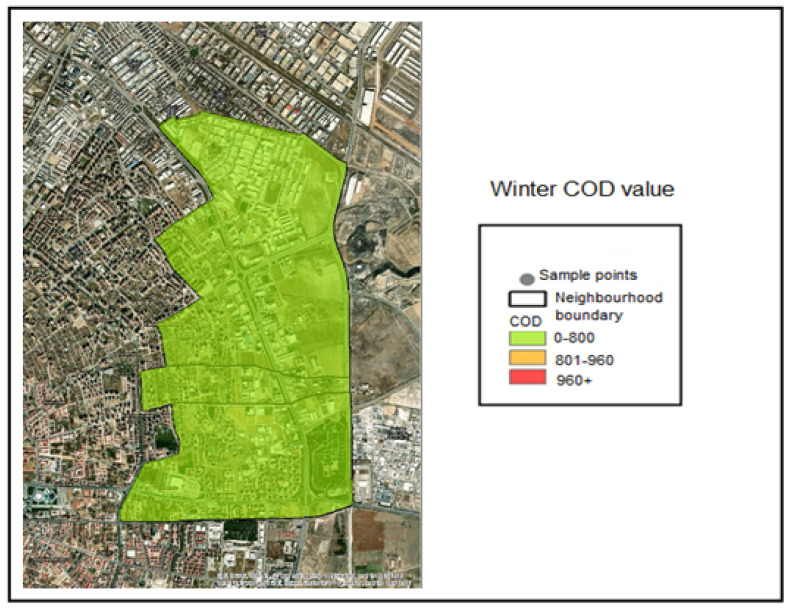
Winter season COD distribution map.

**Figure 8:**
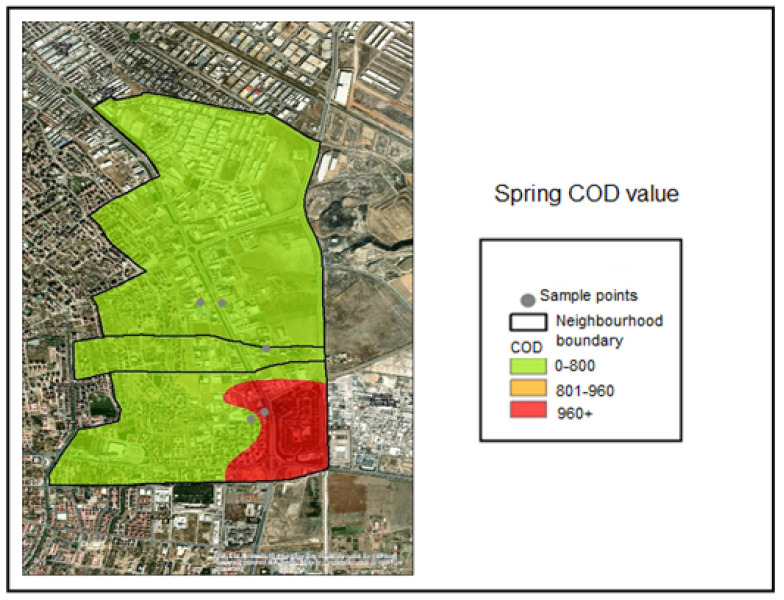
Spring season COD distribution map.

**Figure 9:**
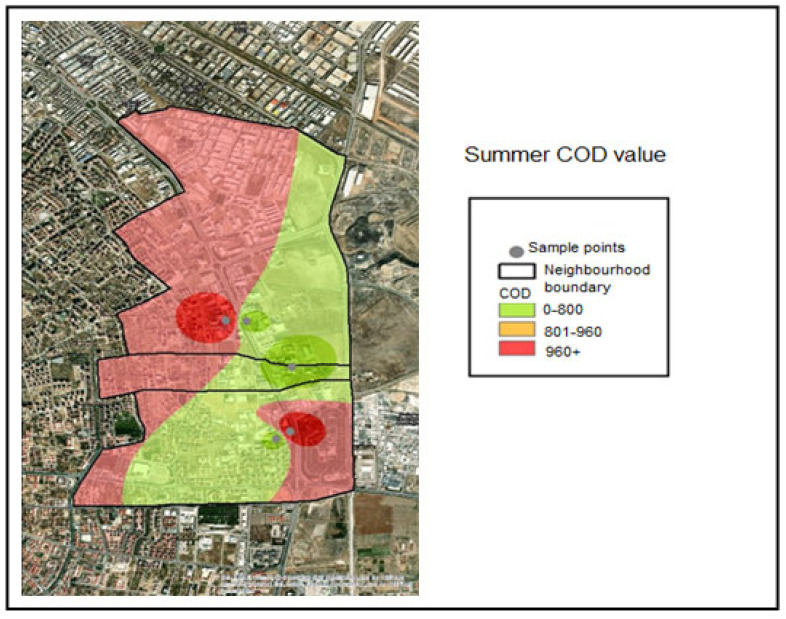
Summer season COD distribution map.

The corresponding color coding was applied based on these classification thresholds. Accordingly, examination of the COD distribution maps indicates that all samples collected during the winter season fell within the compliant range (≤800 mg/L) and were therefore represented in green (Figure 7). During the spring season, the COD concentration measured at Industry 5 exceeded 960 mg/L and was therefore classified as noncompliant (Figure 8). During the summer season, the COD concentrations measured at Industries 4 and 5 exceeded 960 mg/L and were classified as noncompliant, as indicated by red coding (Figure 9).

The suspended solids analyses conducted on the collected samples were classified into the following concentration ranges based on spatial interpolation within the GIS application:

- SS ≤ 400 mg/L—Compliant range (Green);
- 401–480 mg/L—Threshold-adjacent range (Yellow);
- SS > 480 mg/L—Noncompliant (Red).

**Figure 10:**
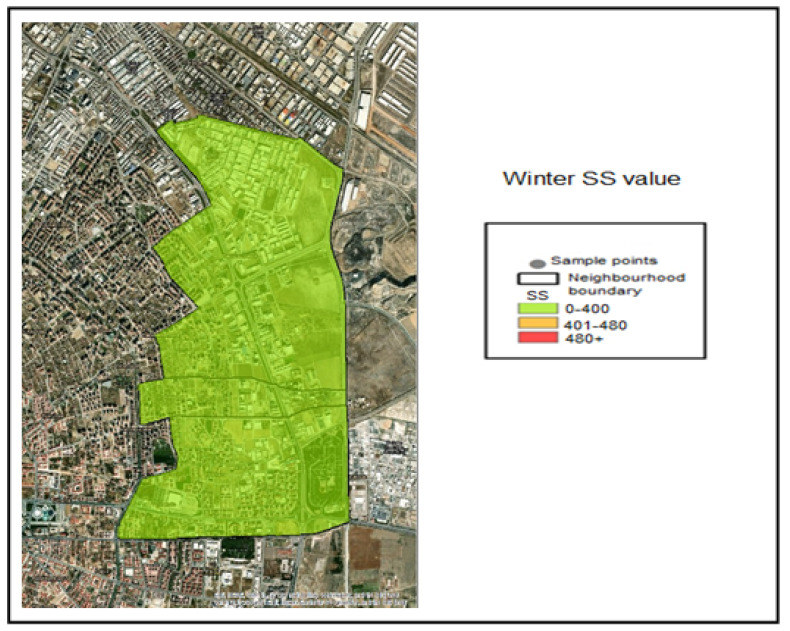
Winter season SS value pollution map.

**Figure 11:**
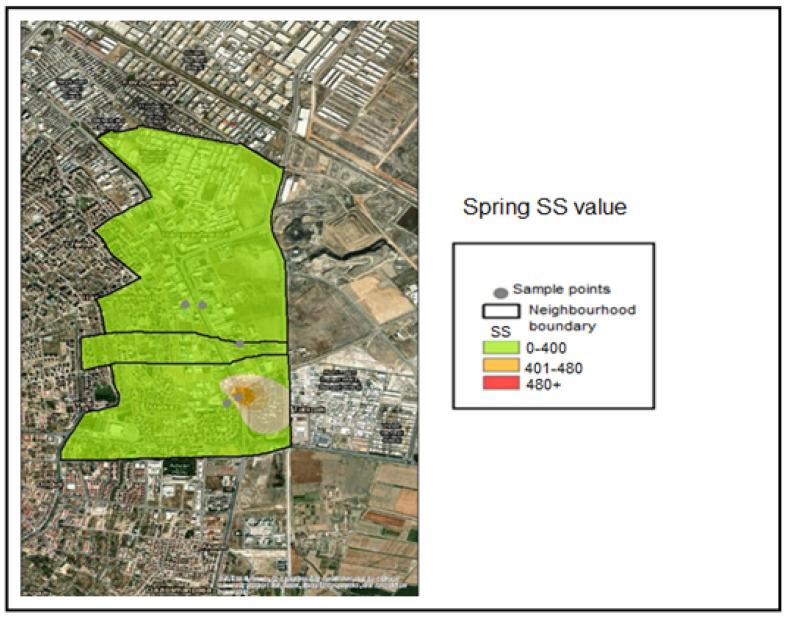
Spring season SS value pollution map.

**Figure 12:**
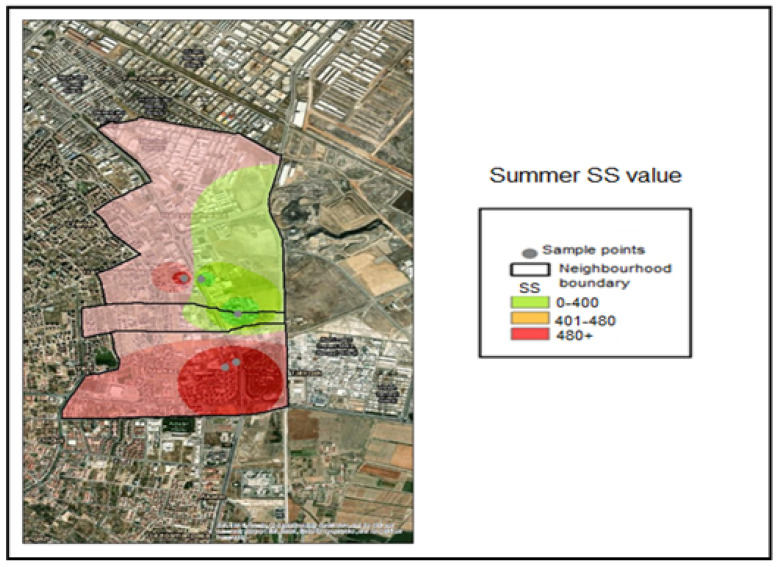
Summer season SS value pollution map.

The corresponding color coding was applied based on these classification thresholds. Accordingly, examination of the SS (Suspended Solid) distribution maps indicates that all samples collected during the winter season fell within the compliant range (≤400 mg/L) and were therefore represented in green (Figure 10). During the spring season, the SS concentration measured at Industry 5 fell within the threshold-adjacent interval (401–480 mg/L) (Figure 11). During the summer season, the SS concentrations measured at Industries 3, 4, and 5 exceeded 480 mg/L and were therefore classified as noncompliant (red) (Figure 12).

The Oil–Grease analysis performed on the samples taken determined the following value ranges through interpolation within the application:

- Oil–Grease ≤ 150 mg/L —Compliant range (Green);
- 151–165 mg/L—Threshold-adjacent range (Yellow);
- Oil–Grease> 165 Noncompliant (Red).

**Figure 13:**
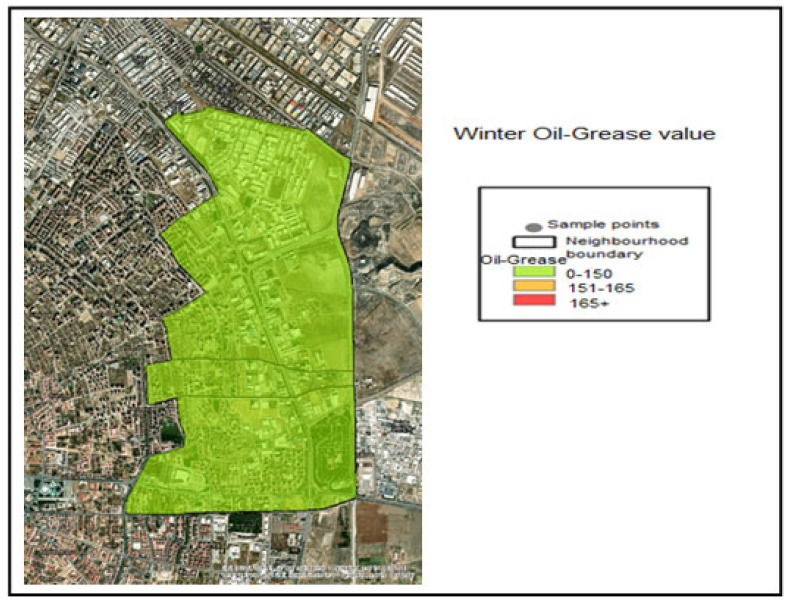
Winter season oil and grease distribution map.

**Figure 14:**
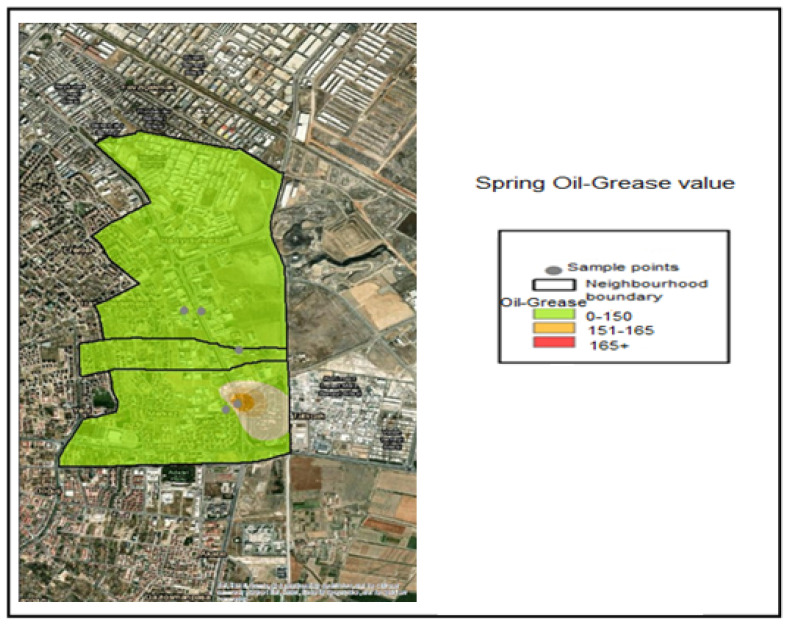
Spring season oil and grease distribution map.

**Figure 15:**
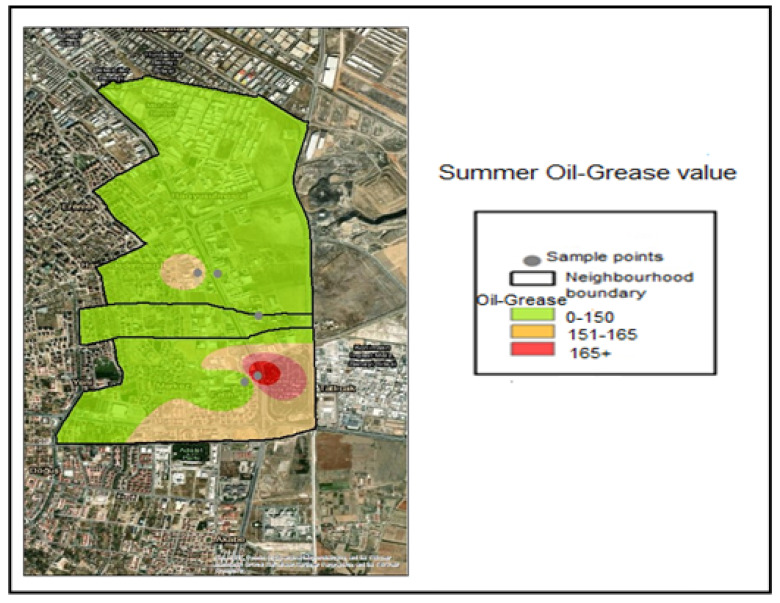
Summer season oil and grease distribution map.

The necessary color coding has been applied. Accordingly, when examining the Oil–Grease value pollution maps, it is seen that the samples taken during the winter months are within the normal range of 0–150 for all industries and are colored green (Figure 13). The spring month Industry 5 Oil–Grease value is in the range of 151–165, which is considered a sensitive level (Figure 14). In samples taken during the summer month, the oil–grease value for Industry 4 is between 151 and 165, which is a sensitive value. For Industry 5, it is 166+, indicating a dirty level (Figure 15).

Industrial wastewater discharged into the municipal sewer system must undergo appropriate pretreatment processes to comply with the applicable discharge standards prior to its release into the receiving environment [21]. Otherwise, such wastewater may adversely affect the treatment performance of municipal wastewater treatment plants, resulting in reduced treatment efficiency and increased in operational costs [22,23]. In addition, insufficiently treated industrial discharges may cause operational problems, such as clogging, corrosion, and odor formation within the municipal sewer system, thereby increasing infrastructure operational costs [24,25]. It has been reported that wastewater discharges from industrial facilities located in close proximity to residential areas, as observed in Konya, may generate significant impacts that adversely affect both urban quality of life and environmental sustainability [26].

Samples are collected at regular intervals from industrial facilities, and monitoring activities are conducted in accordance with the relevant regulatory parameters [27]. However, the increasing number of industrial facilities has rendered the processes of sampling, analysis, and detection of noncompliance progressively more complex and time-consuming [28]. Although Konya Metropolitan Municipality has integrated various urban datasets into a Geographic Information System (GIS) platform as part of its smart city applications, a comprehensive GIS-based monitoring system specifically dedicated to industrial wastewater has not yet been established, despite industrial discharges constituting one of the primary environmental pressures contributing to increased sewer load [29,30].

Today, many cities utilize smart data infrastructures and GIS-based decision-support systems to manage urban challenges in a more comprehensive, systematic, and efficient manner.These systems are actively employed across a wide range of municipal services and continue to be expanded to support emerging application areas [31,32]. Within the scope of this study, it is proposed that the analytical results be transferred to a GIS environment to enable comprehensive monitoring and spatial evaluation of the pollution load associated with samples collected from sewer connection points of industrial facilities located in the selected pilot area. Within this preliminary framework, the sample analysis results obtained from the Hacıyusufmescit, Emirgazi, and Fetih neighborhoods were visualized in both temporal and spatial dimensions using ArcGIS software (Esri, Redlands, CA, USA), and their effects on the sewer system were evaluated through spatial pattern analysis.

As part of the study, samples collected from selected industrial facilities were analyzed for pH, COD, SS, and oil and grease parameters. The resulting data were subsequently transferred to the ArcGIS environment and spatially mapped. The generated maps revealed monthly variations in parameter concentrations, indicating potential inconsistencies in wastewater control and pretreatment practices at certain industrial facilities. To obtain more reliable and representative results, sampling was repeated at different time intervals, resulting in multiple spatial distribution maps that reflect temporal variability. In cases where pretreatment systems were not implemented or were not operated effectively, areas exhibiting parameter concentrations exceeding regulatory limits were identified through spatial mapping.

A comprehensive evaluation of the sample results and the maps generated for the pilot area was conducted within the ArcGIS environment; the resulting data were monitored in both temporal and spatial dimensions, and areas exerting potential pollution pressure were identified through spatial pattern analysis. This approach demonstrates that GIS-based analyses can be effectively utilized as a decision-support tool for monitoring and managing pollution sources associated with the municipal sewer system [33].

The results obtained from samples collected at sewer junction points revealed elevated COD and SS concentrations (Table 7). Similarly elevated concentrations detected in samples collected from industrial facilities suggest that these industrial discharges may constitute a significant contributing factor to the odor problems observed in the region. These findings highlight that accurate characterization of wastewater discharged into the municipal sewer system from the relevant area represents a critical factor for the effective and sustainable operation of urban wastewater treatment plants [34,35]. Furthermore, it is considered that the aforementioned industrial discharges may adversely affect operational performance by inducing sudden load fluctuations within the municipal sewer system and wastewater treatment plants [36].

It was determined that food industry facilities are concentrated within the study area and that all examined facilities belong to this sector. Therefore, monitoring heavy metal parameters was not prioritized, and the detection of very low heavy metal concentrations in samples collected from sewer junction points supported this approach (Table 7). This research represents a pilot-scale application covering three neighborhoods and five industrial facilities. However, the findings are significant in terms of facilitating the integration of industrial wastewater monitoring data across Konya into a GIS-based system by the Konya Water and Sewerage Administration General Directorate (KOSKİ).

The ability to collect sample data from industrial facilities on a single digital platform and monitor these data through GIS-based maps may provide significant contributions to the early detection and comprehensive assessment of environmental problems, such as odor and pollution, across the city. Furthermore, it may support more efficient operation of the municipal sewer system, reduction in operational costs, systematic execution of control processes, and acceleration of decision-making mechanisms [37]. In this regard, the GIS-based monitoring approach is considered to enable the development of a proactive and data-driven management model for urban wastewater management [38,39].

## 3. Materials and Methods

### 3.1. Determination of the Study Area

**Figure 16:**
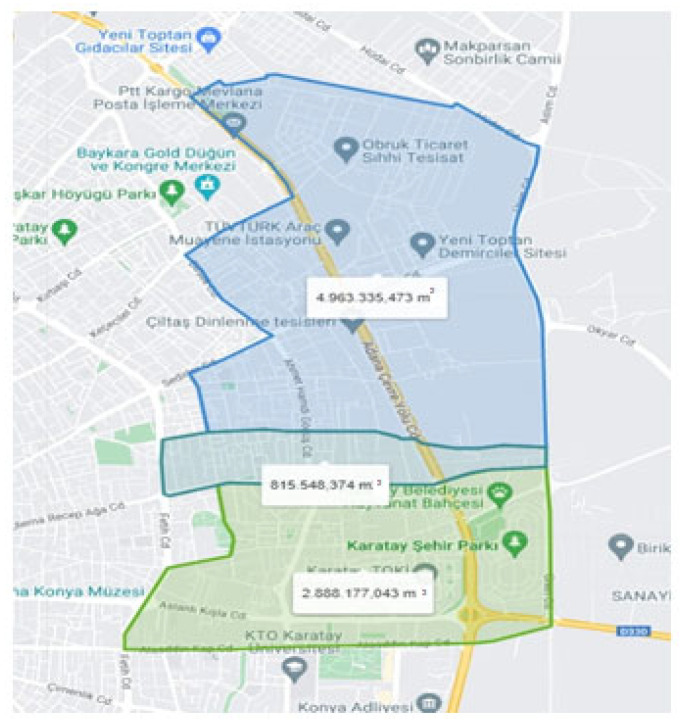
Hacıyusufmescit, Emirgazi, and Fetih neighborhoods [1].

**Figure 17:**
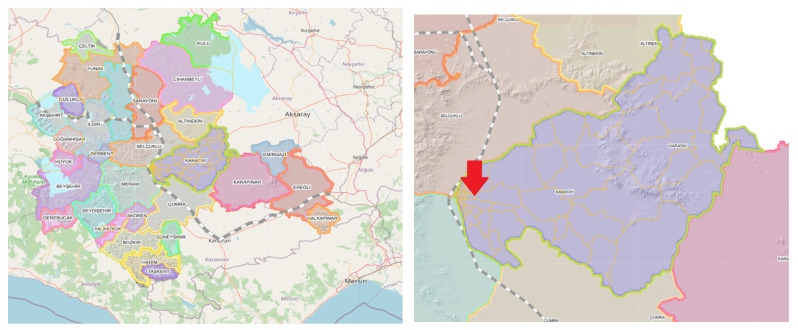
Karatay District [1].

The study area was defined as the region encompassing the Hacıyusufmescit, Emirgazi, and Fetih neighborhoods (Figure 16) within the Karatay district (Figure 17) of Konya province. The region comprises both residential areas and industrial facilities. The close proximity of residential areas to industrial facilities constituted a significant criterion in the selection of the study area. Furthermore, odor-related problems and associated public complaints are concentrated in this region at the provincial level. This consideration constituted an additional justification for the selection of the study area. The study area is served by a combined sewer system through which stormwater, domestic wastewater, and industrial wastewater are conveyed to the municipal wastewater treatment plant. Accordingly, samples were collected from sewer system junction points in addition to those obtained from industrial facilities.

**Figure 18:**
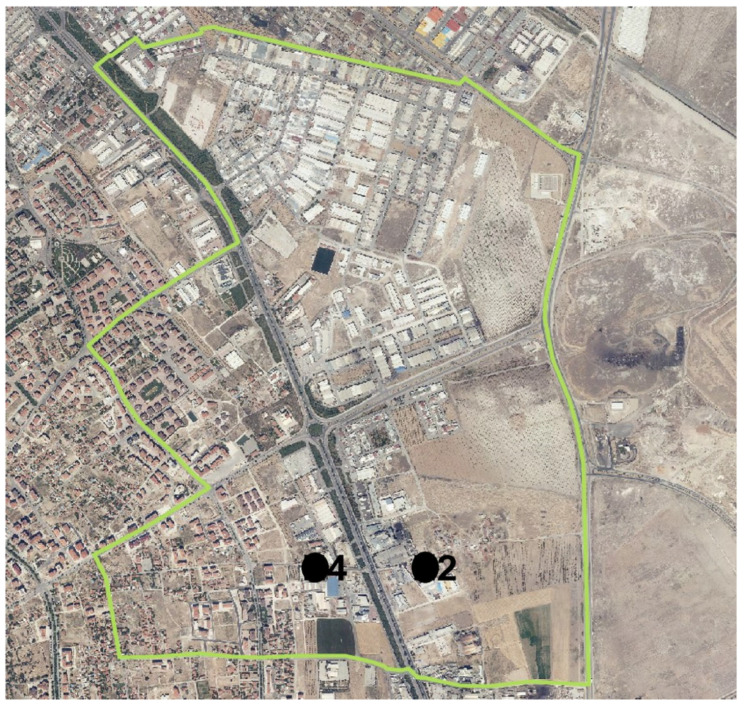
Hacıyusufmescit neighborhood and monitoring points. 2: Industry 2; 4: Industry 4.

Samples were collected from two industrial facilities identified in the Hacıyusufmescit neighborhood monitoring points (Figure 18).

**Figure 19:**
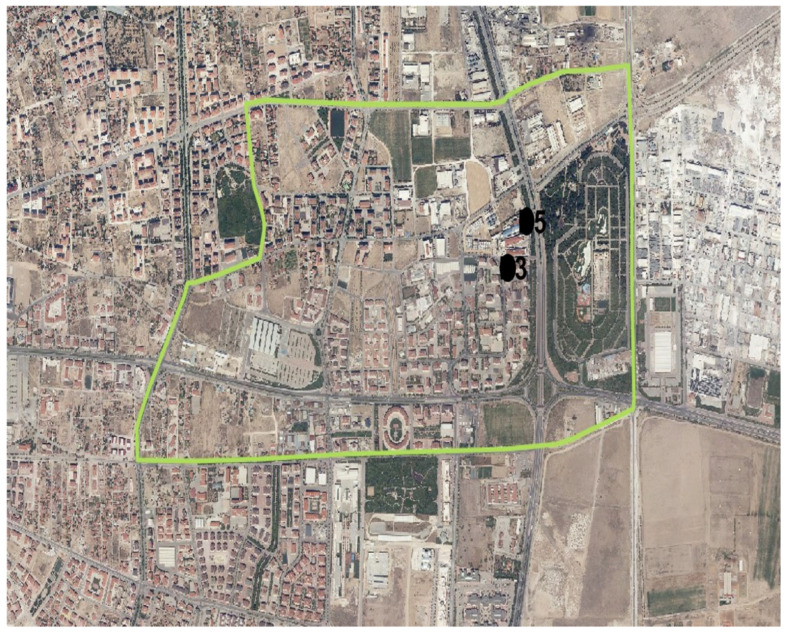
Fetih neighborhood and monitoring points. 3: Industry 3; 5: Industry 5.

In the Fetih neighborhood, samples were collected from two designated industrial facilities monitoring points (Figure 19).

**Figure 20:**
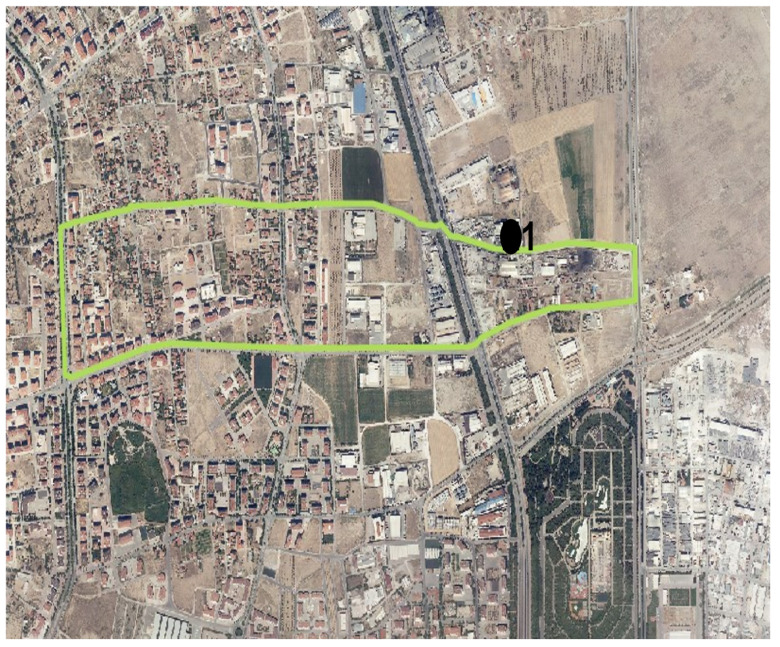
Emirgazi neighborhood and monitoring points. 1: Industry 1.

In the Emirgazi neighborhood, samples were collected from one industrial facility monitoring point (Figure 20).

**Figure 21:**
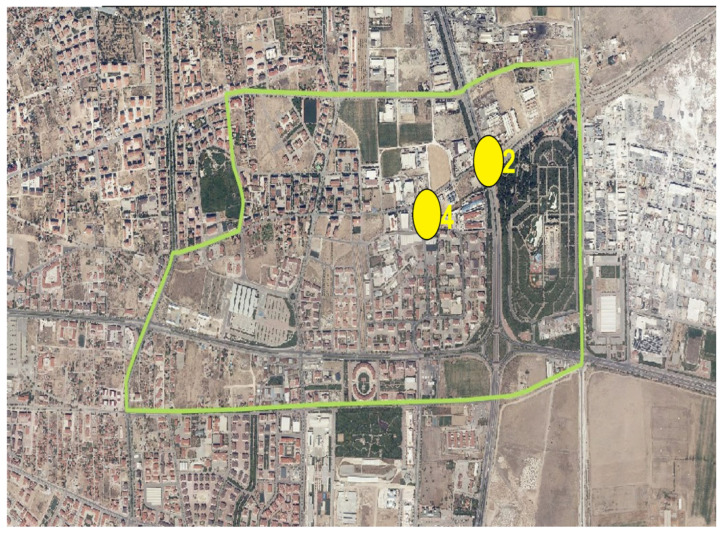
Fetih neighborhood sewer monitoring points. 2: Sewer 2.; 4: Sewer 4.

Wastewater samples were collected from two sewer junction points identified in the Fetih neighborhood sewer monitoring points (Figure 21).

**Figure 22:**
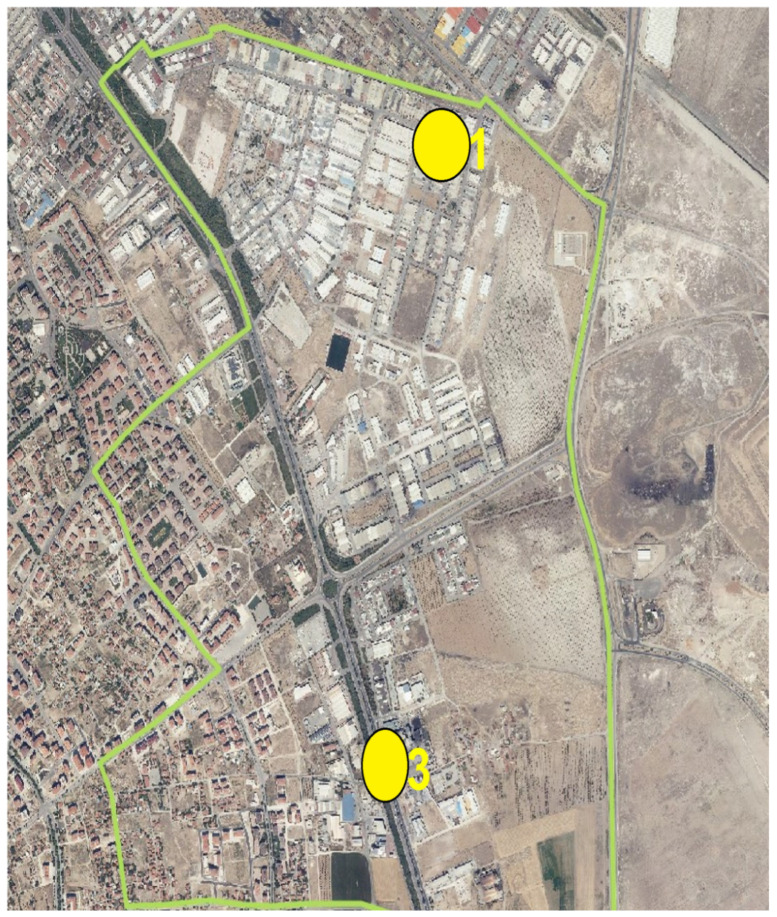
Hacıyusufmescit neighborhood sewer monitoring points. 1: Sewer 1; 3: Sewer 3.

Wastewater samples were collected from two sewer junction points identified in the Hacıyusufmescit neighborhood sewer monitoring points (Figure 22).

### 3.2. Sample Collection and Analysis Processes

**Figure 23:**
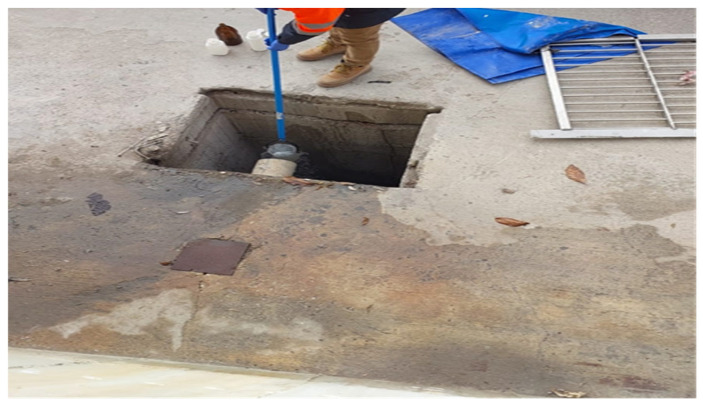
Sampling with a sampling rod [1].

Seasonal samples were collected from the facilities shown in Figure 18, Figure 19, Figure 20, Figure 21 and Figure 22. The samples were collected as instantaneous samples using a sampling probe (Figure 23). The analyses were conducted at the Konya Wastewater Treatment Plant Laboratory and at laboratories authorized by the Ministry of Environment, Urbanization, and Climate Change and accredited by TÜRKAK.

### 3.3. Stages of Temporal and Spatial Analysis in a GIS Environment

**Figure 24:**
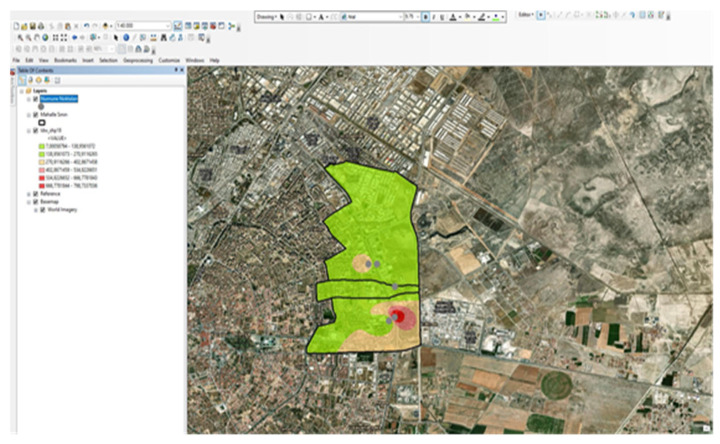
ArcToolbox-IDW Interpolation menu [1].

The first step in the spatial analysis phase is the creation of a base map. For the map to be created, neighborhood boundaries obtained from the NetCAD 8.0 program via the Software Branch Directorate of the KOSKİ Information Processing Department were imported into ArcGIS. After the sample results were entered into Excel, all data was converted to XLS format. The neighborhood boundaries were created in a format compatible with the ArcGIS database. The location data for industrial facilities was obtained from the KOSKİ GIS and entered into Excel along with the sample results. It was overlaid onto the base map using the Open Attribute Table tab. The map was then created by entering the necessary information in the IDW (Inverse Distance Weighted Interpolation Method) Interpolation menu under the ArcToolbox tab (Figure 24).

## 4. Conclusions

The findings of this study indicate that the research area is predominantly characterized by food industry facilities, with all examined sites belonging to this sector. This situation led to the monitoring of heavy metal parameters not being considered a priority; indeed, the very low levels of heavy metal concentrations measured confirmed this approach. Although the results were obtained from a pilot-scale application covering three neighborhoods and five industrial facilities, they offer important insights regarding the integration of a GIS-based wastewater monitoring system within the Konya Wastewater Administration General Directorate (KOSKİ).

The need for continuous spatial analysis is of critical importance, particularly in processes such as the detection of illegal discharges, the identification of pollutant dispersion caused by combined sewer overflows, and the assessment of the impacts of infrastructure leaks. In this context, it has been determined that spatial interpolation techniques integrated with GIS enable a more accurate and comprehensive representation of pollution distribution by converting limited and sparse sampling data into continuous surfaces [40,41].

The ability to collect sample data from industrial facilities through a single digital platform provides critical contributions to the early detection and comprehensive assessment of environmental issues (such as odor and pollution) at the urban scale. Additionally, this approach enables more effective coordination in wastewater management processes, accelerates decision-making mechanisms, and facilitates the development of data-driven management strategies. In this context, it is assessed that the GIS-based monitoring approach can significantly contribute to the development of a proactive and data-driven management model in urban wastewater management.

## Figures and Tables

**Table 2:** Sampling results for Industry 1.

Parameters (mg/L)	Winter (Mean Value)	Spring (Mean Value)	Summer (Mean Value)
pH	6.42	6.58	7.26
COD	369.60	585.50	89.60
SS	182.90	196.85	9.20
Oil–Grease	94.40	89.60	18

**Table 3:** Sampling results for Industry 2.

Parameters (mg/L)	Winter (Mean Value)	Spring (Mean Value)	Summer (Mean Value)
pH	7.86	7.01	7.89
COD	432.90	521.62	70.40
SS	353.70	272.60	45.70
Oil–Grease	83.60	88.80	54.20

**Table 4:** Sampling results for Industry 3.

Parameters (mg/L)	Winter (Mean Value)	Spring (Mean Value)	Summer (Mean Value)
pH	7.63	8.07	8.68
COD	98.20	511	400
SS	22.40	279.60	835
Oil–Grease	96.50	116.80	67

**Table 5:** Sampling results for Industry 4.

Parameters (mg/L)	Winter (Mean Value)	Spring (Mean Value)	Summer (Mean Value)
pH	7.45	6.89	5.87
COD	68.20	638.70	15,680
SS	47.40	310.40	860
Oil–Grease	62	101.60	154

**Table 6:** Sampling results for Industry 5.

Parameters (mg/L)	Winter (Mean Value)	Spring (Mean Value)	Summer (Mean Value)
pH	7.41	8.40	6.1
COD	385.4	1216	24,960
SS	133.5	406	2970
Oil–Grease	118	170	254

**Table 7:** Sewer junction sampling results.

Parameters	S1-Winter (Mean Value)	S2-Winter (Mean Value)	S3-Spring (Mean Value)	S4-Spring (Mean Value)
pH	7.93	7.9	7.81	8.4
COD (mg/L)	1370.11	772.41	2776.86	4575.21
SS (mg/L)	232	236	477	2520
Oil–Grease (mg/L)	110	86	-	-
Temperature °C	16.2	16.3	14.3	15.1
Conductivity (mS)	1816	1831	4540	2510
Ammonium nitrogen (NH_4_-N) (mg/L)	52.2	66.3	23.6	78
Nitrate nitrogen (NO_3_^−^N) (mg/L)	2.79	3.93	7.3	6.2
Total Nitrogen (TN) (mg/L)	99.3	103.8	109.2	297
Total Phosphorus (TP) (mg/L)	10.62	11.85	50.22	96.1
Aluminum (Al) (mg/L)	<0.0475	<0.01	0.031	0.532
Cadmium (Cd) (mg/L)	<0.01	<0.01	<0.001	<0.001
Chromium (Cr) (mg/L)	0.8778	0.9053	<0.002	<0.002
Copper (Cu) (mg/L)	0.1125	0.0391	0.0052	<0.002
Iron (Fe) (mg/L)	2.504	0.8929	0.095	0.053
Nickel (Ni) (mg/L)	0.0143	<0.01	<0.004	<0.004
Lead (Pb) (mg/L)	<0.0371	<0.0371	<0.005	<0.005
Zinc (Zn) (mg/L)	0.3817	0.1694	0.216	0.015

S: Sewer junction; S1: Sewage 1; S2: Sewage 2; S3: Sewage 3; S4: Sewage 4; -:has not been analyzed.

## Data Availability

The data are included in the paper.
